# A multi-country comparison of jurisdictions with and without mandatory nutrition labelling policies in restaurants: analysis of behaviours associated with menu labelling in the 2019 International Food Policy Study

**DOI:** 10.1017/S1368980023001775

**Published:** 2023-11

**Authors:** Michael Essman, Thomas Burgoine, Adrian Cameron, Andrew Jones, Monique Potvin Kent, Megan Polden, Eric Robinson, Gary Sacks, Richard D Smith, Lana Vanderlee, Christine White, Martin White, David Hammond, Jean Adams

**Affiliations:** 1 MRC Epidemiology Unit, University of Cambridge, Cambridge, UK; 2 Institute for Health Transformation, Deakin University, Burwood, Australia; 3 School of Psychology, Liverpool John Moore’s University, Liverpool, UK; 4 School of Epidemiology and Public Health, University of Ottawa, Ottawa, Canada; 5 Institute of Population Health Sciences, University of Liverpool, Liverpool, UK; 6 University of Exeter Medical School, Exeter, UK; 7 School of Nutrition, Centre Nutrition, santé et société (NUTRISS), INAF, Université Laval, Québec, Canada; 8 School of Public Health Sciences, University of Waterloo, Waterloo, Canada

**Keywords:** Obesity, Food policy, Behaviour, Menu label, Restaurant

## Abstract

**Objective:**

To examine differences in noticing and use of nutrition information comparing jurisdictions with and without mandatory menu labelling policies and examine differences among sociodemographic groups.

**Design:**

Cross-sectional data from the International Food Policy Study (IFPS) online survey.

**Setting:**

IFPS participants from Australia, Canada, Mexico, United Kingdom and USA in 2019.

**Participants:**

Adults aged 18–99; *n* 19 393.

**Results:**

Participants in jurisdictions with mandatory policies were significantly more likely to notice and use nutrition information, order something different, eat less of their order and change restaurants compared to jurisdictions without policies. For noticed nutrition information, the differences between policy groups were greatest comparing older to younger age groups and comparing high education (difference of 10·7 %, 95 % CI 8·9, 12·6) to low education (difference of 4·1 %, 95 % CI 1·8, 6·3). For used nutrition information, differences were greatest comparing high education (difference of 4·9 %, 95 % CI 3·5, 6·4) to low education (difference of 1·8 %, 95 % CI 0·2, 3·5). Mandatory labelling was associated with an increase in ordering something different among the majority ethnicity group and a decrease among the minority ethnicity group. For changed restaurant visited, differences were greater for medium and high education compared to low education, and differences were greater for higher compared to lower income adequacy.

**Conclusions:**

Participants living in jurisdictions with mandatory nutrition information in restaurants were more likely to report noticing and using nutrition information, as well as greater efforts to modify their consumption. However, the magnitudes of these differences were relatively small.

Eating from out-of-home food outlets is common, is expected to increase globally over the next decade^(1)^, and is associated with poorer dietary quality, increased energy intake and obesity^(2–4)^ Eating food from out-of-home food outlets may lead to weight gain due to larger portion sizes and greater energy density of food from these outlets, which may cause consumers’ energy intake to exceed their energy requirements^(2)^. Furthermore, previous work has found both experts and the general public tend to be poor estimators of their energy intake from restaurants^(5,6)^.

Historically, there have been fewer regulations on labelling the nutritional content of foods purchased at out-of-home food outlets compared to those purchased in grocery stores. One policy response to help inform consumers about the nutrition content of out-of-home eating is to include energy labels on menus. Mandatory energy labelling policies may improve diets through various pathways including informing consumers about the energy content of food options to help them make a more informed selection, shifting food choices towards healthier options, and incentivising the food industry to offer reduced energy versions of their offerings, via reformulation and reduced serving sizes, or introducing new products^(7,8)^. Menu labelling policies are also thought to be cost effective population-level interventions to improve diets, reduce obesity and prevent associated chronic diseases^(9)^. Several recent meta-analyses of evaluations of menu labelling interventions found that, although study quality tends to be mixed, energy labels may lead to small reductions in energy intake among adults at a population level, and energy labelling may reduce the amount of energy consumers purchase from restaurants^(10–12)^.

Although there is a vast literature on the effects of menu labelling on behaviour, to our knowledge no studies have examined the effects of implemented menu labelling policies in a multi-country context. Several reviews have been published examining the impact of menu labelling policies in ‘real world’ and laboratory settings^(7,10,12–18)^. However, the majority of studies are small randomised controlled trials, include populations only from the USA or were implemented in small settings such as a university cafeteria. Evidence from the USA suggests energy labelling can lead to small improvements in fast food meal quality and small-to-moderate decreases in energy purchased from supermarkets and fast food restaurants, but reductions in purchases may diminish over time^(19–21)^. Many studies also lack a comparison group to examine the effects of menu labelling policies^(22)^ and those that do are limited to analyses of individual food service chains or examine policy in individual cities, potentially limiting generalisability of findings^(23–25)^. Although a randomized controlled trial (RCT) is typically assumed to have less risk of confounding than observational studies, it is essential to also understand the effects of policies implemented in the real world, particularly in a large state-wide or even national setting, where ‘real world’ effectiveness may differ from efficacy in a RCT. In the context of national-level diet surveys, many are conducted too infrequently to be compared with other countries during the same time period or they may capture different diet-related behaviours, which limits comparability^(26,27)^. Thus, a multi-country approach to evaluating the impacts of food policies addresses current gaps in national monitoring surveys^(27)^.

Mandatory menu labelling has been implemented in national and subnational jurisdictions^(28)^, and this study presents a population-based evaluation to clarify the impacts of real world menu labelling policies. We utilised data from 2019 of the International Food Policy Study (IFPS), a multi-country repeated cross-sectional survey of five upper- and middle-income countries including Australia, Canada, Mexico, the United Kingdom, and the USA. The IFPS allows for comparisons of polices in countries or jurisdictions that have implemented compared to those that have not implemented^(27)^. The five countries in the IFPS have varying mandatory menu labelling regulations, with some policies mandatory at the national levels, others at the state/province level and others with no mandatory menu labelling regulations. Thus, this multi-country survey includes large populations that were and were not exposed to mandatory energy labelling regulations at the time of data collection.

The purpose of this study was to examine the prevalence of noticing and using menu labels and the behaviours associated with menu labelling overall and by sociodemographic characteristics, comparing jurisdictions with and without mandatory menu labelling policies. The first research question was whether there were any significant differences in these behavioural outcomes according to policy status. We hypothesised that jurisdictions with mandatory menu labelling policies would have higher rates of noticing and use of menu labels compared to jurisdictions without. The second research question was whether differences by policy status varied for sociodemographic groups. Given the high agency requirement of menu labelling policies, we hypothesised that variations would exist across sociodemographic groups.

## Methods

### Dataset

Data are from the 2019 wave of the IFPS, a multi-country repeat cross-sectional study of eating patterns and policy-relevant behaviours, and include data from Australia, Canada, Mexico, the United Kingdom and the USA^(27,29)^. These countries were selected for the IFPS survey because of differences in food-related policies prior to the first wave and the potential for change in policies between subsequent waves (Table 1). The study sample was recruited from Nielsen Consumer Insights Global Panel, which provides standardised recruitment sampling across countries. A random sample of participants aged 18–99 from the Nielsen Consumer Insights Global Panel and their partners’ panels were invited by email to complete the IFPS survey^(29)^. Online surveys were completed between November and December of 2019. Questionnaires and further details about recruitment are available from the IFPS: Technical Report 2019^(29)^.

**Table 1:** Categorisation of jurisdictions according to presence or absence of mandatory menu labelling policies before 2019 data collection^(28)^

Country and policy status	Jurisdiction and year	Policy description
Australia – Policy Present	Five states/territories: Australian Capital Territory (2013), New South Wales (2011), Queensland (2016), South Australia (2012), Victoria (2018)	Energy content must be presented in restaurant chains with at least twenty state outlets or fifty nationwide outlets
Canada – Policy Present	One province: Ontario (2017)	Menu labelling requirements for all food premises with > twenty locations, with the Healthy Menu Choices Act (HMCA)
United States – Policy Present	National policy fully implemented in 2017 (introduced in 2014)	National menu labelling requirements were announced in 2010 as part of the Affordable Care Act and officially came into force on 7 May 2017. Large chain restaurants (with twenty or more locations) are required to include calorie counts to their menus and menu boards
Australia – No Policy	Western Australia, Tasmania, Northern Territory	Voluntary implementation
Canada – No Policy	All provinces other than Ontario	
Mexico – No Policy	N/A	Packaged foods require warning labels, but there are no restaurant menu labelling laws.
United Kingdom – No Policy	N/A	In April 2022, England introduced mandatory calorie menu labelling for large out-of-home food businesses with more than 250 employees, but these policies were not implemented at the time of data collection for this study

### Exposure

Policy status was treated as an indicator variable for either Policy Present or No Policy in the analysis, and Policy Present was defined as having a mandatory menu labelling policy in place during 2019. Table 1 shows the jurisdictions included in this study that did and did not have a mandatory menu labelling policy implemented during 2019. The Policy Present group includes the USA and jurisdictions of Australia and Canada with mandatory labelling regulations (Table 1). In April 2022, England introduced mandatory calorie menu labelling for large out-of-home food businesses, defined as those with more than 250 employees^(30)^; however, at the time of the data collection for this study, England and Mexico had not implemented menu labelling requirements and served as ‘comparison’ conditions. The No Policy group includes Mexico, the United Kingdom, and segments of Australia and Canada without mandatory labelling regulations (Table 1). We separated regions with mandatory labelling policies from areas without in both Canada and Australia. Participants in Canada answered ‘What province or territory do you live in?’ and participants in Australia answered ‘What state or territory do you live in?’ For Canada, responses of Ontario were coded to Policy Present, and all provinces other than Ontario were coded to No Policy (Table 1). For Australia, responses of Australian Capital Territory, New South Wales, Queensland, South Australia and Victoria were coded to Policy Present, and responses of Western Australia, Tasmania, Northern Territory were coded to No Policy (Table 1).

### Outcomes

There are myriad ways in which consumers make food-related decisions. For example, contemporary behaviour change theory conceptualises behaviour as a result of interacting capability, opportunity and motivation^(31)^. Price, taste and convenience are also key factors in making food decisions. Other potential psychological mechanisms are involved in eating behaviour status quo bias – people eat what is typical and available such as large restaurant portion sizes – simplicity and energy compensation^(32)^. The conceptual framework used in the present study assumes in order to make eating decisions, nutrition information must be noticed, then used, and finally used in a particular way. Previous work has examined the rates at which consumers notice and use nutrition information^(24)^. This study examines several self-reported outcomes related to how mandatory menu labelling policies are theorised to affect behaviours associated with menu labelling. Outcomes measured were as follows: *noticing nutrition labels*, *use of nutrition labels*, *ordered something different*, *ate less of the food they ordered*, *visited different restaurants* or *ate at restaurants less often*. These measures, as well as sociodemographic characteristics, are defined in Table 2, including the survey questions and coding for the analysis. Responses to *noticed nutrition information* and *used nutrition information* questions refer to the last time the participant visited a restaurant. Reponses to the behavioural impact of labelling questions refer to behaviours that occurred within the last 6 months and were preceded by the question ‘In the past 6 months, have you done any of the following because of nutrition information in restaurants? (Select all that apply)’ (Table 2). These measures were adapted from previously validated measures and published research^(33)^.

**Table 2:** IFPS 2019 survey questions and variable categorisation

		Response options	
Concept	Item wording (where applicable)	All	Variable coding
Outcomes			
Noticed nutrition information	The last time you visited a restaurant, did you notice any nutrition information?	No, Don’t know, Refuse to answer	No
		Yes	Yes
Used nutrition information	Did the nutrition information influence what you ordered?	No, Don’t know, Refuse to answer	No
		Yes	Yes
Impact of labelling	In the past 6 months, have you done any of the following because of nutrition information in restaurants? (Select all that apply)		
Ordered something different	Ordered something different	Unselected/left blank	No
		Selected	Yes
Ate less of order	Eaten less of the food you ordered	Unselected/left blank	No
		Selected	Yes
Changed restaurant visited	Changed which restaurants you visit	Unselected/left blank	No
		Selected	Yes
Ate at Restaurants Less Often	Eaten at restaurants less often	Unselected/left blank	No
		Selected	Yes
Sociodemographic characteristics			
Sex	What sex were you assigned at birth, meaning on your original birth certificate?	Female	Female
		Male	Male
Age	How old are you?	Numeric: 18–100	18–24
			25–34
			35–44
			45–54
			55–64
			65–74
			75+
Ethnicity	Which of the following best describes your ethnic or racial background?	Country-specific racial and ethnic backgrounds	Minority
			Majority
Education	What is the highest level of education you have completed?	Below upper secondary/high school completion or lower)	Low
		Upper secondary/some post-high school qualifications	Medium
		Tertiary/university degree or higher	High
Income adequacy	Thinking about your total monthly income, how difficult or easy is it for you to make ends meet?	Neither easy nor difficult, Difficult, Very difficult, Don’t know, Refuse to answer	Not Easy
		Easy, Very Easy	Easy

### Sociodemographic characteristics

Sociodemographic characteristics including age, sex, education, income adequacy and ethnicity were included as potential confounders in models. The wording, responses and categories used in analysis of covariates are described in Table 2. Age was categorised into 10-year age brackets, except for the youngest group which included participants aged 18–24 years (Table 2). Because this is a multi-country survey with diverse ethnicities, the most comparable ethnicity measure across all countries was comparing the majority ethnicity to combined minority ethnicities. For income, we used income adequacy as it is associated with economic resources and health and allows for comparability across the multiple countries of the IFPS^(34)^.

### Statistical analysis

All statistical analyses were conducted using Stata, Version 16. Data were weighted with post-stratification sample weights constructed using a raking algorithm with country-specific population estimates from census data based on age group, sex, region, ethnicity (except in Canada) and education (except in Mexico). A detailed explanation of survey weights can be found at http://foodpolicystudy.com/methods (IFPS: Technical Report 2019). Sample weights were used throughout the analysis to minimise the influence of differential non-response and selection bias on the representativeness of findings.

There were 20 968 observations in the dataset. Four of the six behavioural survey questions asked about consumer behaviour at restaurants within the previous six months. Therefore, we restricted our sample size to only those participants who visited a restaurant in the previous six months, reducing the sample size to 19 617. Ethnicity data was missing for 176 observations, and a further forty-eight observations were missing education data and were dropped from the analysis, leaving a complete case analysis sample size of 19 393.

Descriptive statistics were used to summarise the outcomes and sociodemographic characteristics of the sample by policy status. To assess whether there were any significant differences in the six binary outcome measures according to policy status, weighted estimates were calculated using a survey-adjusted logistic regression model for each outcome. Policy status was included as an indicator variable (0 = no policy, 1 = policy), and models were adjusted for covariates selected *a priori*: age, sex, education, income adequacy and race/ethnicity. The differences by policy status for behavioural outcomes were reported as OR with 95 % CI, and statistical differences between policy status groups were tested using Wald tests. Results are also presented as predicted probabilities for all behavioural responses calculated using the margins command in Stata^(35)^, as marginal effects can aid interpretation of magnitude and are more comparable across populations than OR^(36,37)^. Predicted probabilities are the probability that the outcome will occur, estimated by the model. Differences in predicted probabilities were calculated using pairwise comparisons of margins^(37,38)^.

To assess whether differences in outcomes by policy status varied across sociodemographic groups, we added two-way interactions between policy and each sociodemographic variable of interest to logistic regression models. Predicted probabilities of all six outcomes were estimated for each level of demographic variable by policy status. Differences by policy status at all levels of each sociodemographic variable were tested using pairwise comparisons of margins^(37,38)^.

## Results

Table 3 describes the sample characteristics, stratified by policy status for the 19 393 participants analysed. The majority of the sample reported high education, majority ethnicity and not easy income adequacy (i.e. not easy to make ends meet). The distribution of education varied by policy status, with more participants reporting High education in No Policy jurisdictions and more participants reporting Low and Medium education in Policy Present jurisdictions (Table 3).

**Table 3:** Sample demographic characteristics by policy status (unweighted *n*; weighted %)

Variable	No policy (*n* 10 737)		Policy present (*n* 8656)		Total (*n* 19 393)	
	*n*	%	*n*	%	*n*	%
Sex % (*n*)						
Male	5335	48·7	4252	48·9	9587	48·8
Female	5402	51·3	4404	51·1	9806	51·2
Age % (*n*)						
18–24	1259	11·9	799	11·8	2058	11·9
25–34	2411	21·8	1657	18·6	4068	20·3
35–44	1938	17·9	1495	17·4	3433	17·7
45–54	1911	16·0	1234	14·3	3145	15·2
55–64	1573	18·0	1895	21·0	3468	19·4
65–74	1369	12·1	1253	13·4	2622	12·7
75+	276	2·5	323	3·5	599	3·0
Ethnicity % (*n*)						
Majority	9225	83·3	6710	69·7	15 935	77·1
Minority	1512	16·8	1946	30·3	3458	22·9
Income adequacy % (*n*)						
Not Easy (to make ends meet)	7257	70·7	5395	65·6	12 652	68·4
Easy (to make ends meet)	3480	29·3	3261	34·4	6741	31·6
Education % (*n*)						
Low	2789	37·2	2690	47·3	5479	41·8
Medium	2667	21·6	2545	21·9	5212	21·8
High	5281	41·3	3421	30·7	8702	36·5
Countries						
Australia	466	4·8	3387	38·2	3853	20·0
Canada	2519	22·0	1328	17·3	3847	19·9
Mexico	4047	38·4	–		4047	20·9
United Kingdom	3705	34·8	–		3705	19·0
United States	–		3941	44·5	3941	20·3

### Noticing and using nutrition information and changes in behaviours by policy status

Participants in jurisdictions with policies were more likely to *notice nutrition information* compared to jurisdictions without policies (OR = 1·67 (95 % CI 1·53, 1·83)). The predicted probability of noticing nutrition information was 21·2 % (20·2–22·1 %) in jurisdictions with mandatory policies compared to 13·9 % (13·1–14·7 %) in jurisdictions without mandatory menu labelling policies, a significant difference of 7·3 % (6·0–8·6 %) (*P* < 0·001). Participants in jurisdictions with policies were more likely to *use nutrition information* compared to jurisdictions without policies (OR = 1·56 (95 % CI 1·38, 1·76)). The predicted probability of *used nutrition information* was 10·6 % (9·9–11·3 %) in jurisdictions with mandatory policies compared to 7·1 % (6·5–7·7 %) in jurisdictions without mandatory menu labelling policies, a significant difference of 3·5 % (2·5–4·4 %) (*P* < 0·0001) (Table 4).

**Table 4:** Predicted probability weighted estimates for noticing, using and behaviour change from menu labels by policy status in 2019 (*n* 19 393)

Noticed nutritional information	OR	95 % CI	Predicted probability %	95 % CI	*P*
Total Sample	–		17·2	16·6, 17·8	
No Policy	(Ref)		13·9	13·1, 14·7	
Policy Present	**1·67**	**1·53, 1·83**	21·2	20·2, 22·1	
Difference	–		**7·3**	**6·0, 8·6**	*P* **< 0·0001**
Used nutritional information					
Total sample	–		8·7	8·3, 9·2	
No policy	(Ref)		7·1	6·5, 7·7	
Policy present	**1·56**	**1·38, 1·76**	10·6	9·9, 11·3	
Difference	–		**3·5**	**2·5, 4·4**	*P* **< 0·0001**
Ordered something different					
Total sample	–		18·9	18·3, 19·5	
No policy	(Ref)		17·9	17·0, 18·7	
Policy present	**1·17**	**1·07, 1·28**	20·2	19·3, 21·2	
Difference	–		**2·3**	**1·0, 3·7**	*P* **= 0·0005**
Ate less of order					
Total sample	–		11·5	11·0, 12·0	
No policy	(Ref)		10·1	9·4, 10·8	
Policy present	**1·37**	**1·23, 1·52**	13·2	12·4, 14·0	
Difference	–		**3·1**	**2·1, 4·2**	*P* **< 0·0001**
Changed restaurant visited					
Total sample	–		7·5	7·1, 7·9	
No policy	(Ref)		7·0	6·4, 7·6	
Policy present	**1·18**	**1·04, 1·35**	8·1	7·5, 8·7	
Difference	–		**1·1**	**0·2, 2·0**	*P* **= 0·013**
Ate at restaurants less often					
Total sample	–		14·9	14·3, 15·5	
No policy	Ref		15·2	14·4, 16·0	
Policy present	0·95	0·86, 1·04	14·5	13·7, 15·4	
Difference	–		−0·6	–1·8, 0·5	

All models adjusted for age, sex, education, ethnicity, income adequacy.

Bolded values are statistically significant at the 0·05 level.

Participants in jurisdictions with policies were more likely to *order something different* compared to jurisdictions without policies (OR = 1·17 (1·07, 1·28)). The predicted probability of ordering something different as a result of nutrition information was 20·2 % (19·3–21·2 %) in jurisdictions with mandatory policies compared to 17·9 % (17·0–18·7 %) in jurisdictions without mandatory menu labelling policies, a significant difference of 2·3 % (1·0–3·7 %) (*P* = 0·0005). Participants in jurisdictions with policies were more likely to *eat less of their order* compared to jurisdictions without policies (OR = 1·37 (1·23, 1·52)). The predicted probability of *ate less of order* was 13·2 % (12·4–14·0 %) in jurisdictions with mandatory policies compared to 10·1 % (9·4–10·8 %) in jurisdictions without mandatory menu labelling policies, a significant difference of 3·1 % (2·1–4·2 %) (*P* < 0·0001). Participants in jurisdictions with policies were more likely to *change restaurant visited* compared to jurisdictions without policies (OR = 1·18 (1·04, 1·35)). The predicted probability of *changed restaurant visited* was 8·1 % (7·5–8·7 %) in jurisdictions with mandatory policies compared to 7·0 % (6·4–7·6 %) in jurisdictions without mandatory menu labelling policies, a significant difference of 1·1 % (0·2–2·0 %) (*P* = 0·013) (Table 4). There was no significant difference in the odds of *ate at restaurants less often* between jurisdictions with and without mandatory menu labelling policies. We also examined the differences in outcomes by country and policy status descriptively, finding similar patterns of greater noticing, use and behavioural outcomes associated with menu labelling in Policy Present jurisdictions (online Supplementary Table 1).

### Interaction results: differences in behaviour by sociodemographic characteristics

Next, we examined whether differences between policy groups varied by sociodemographic characteristics. Only those models with significant interactions are presented below in Tables 5–7. For *noticed nutrition information*, the greatest difference between policy groups was seen for 55–64 year olds (difference of 12·6 %, 95 % CI 9·9, 15·4; *P* < 0·001) and 65–74 year olds (difference of 9·6 %, 95 % CI 6·5, 12·8; *P* < 0·001) (Table 5). These differences were primarily due to lower rates of *noticed nutrition information* for those age groups in No Policy jurisdictions. There was a significantly greater difference between policy groups for high education (10·7 %, 95 % CI 8·9, 12·6) compared to low education (4·1 %, 95 % CI 1·8, 6·3; *P* < 0·001) participants (Table 5). For *use nutrition information*, the greatest differences between policy groups were again for the oldest groups: with a difference of 4·9 % (95 % CI 2·9, 7·0; *P* < 0·001) for 55–64 year olds and a difference of 4·8 % (95 % CI 2·7, 6·8; *P* < 0·001) for 65–74 year olds (Table 5). There was a significantly greater difference between policy groups for high education (4·9 %, 95 % CI 3·5, 6·4) compared to low education (1·8 %, 95 % CI 0·2, 3·5) participants (*P* = 0·006) (Table 5).

**Table 5:** Predicted probability weighted estimates – % (95 % CI) – for models tested with interaction between policy and sociodemographic variables

	Noticed nutrition information						Used nutrition information					
	No policy%	95 % CI	Policy present%	95 % CI	Difference%	95 % CI	No policy	95 % CI	Policy present%	95 % CI	Difference%	95 % CI
Age												
18–24	19·6	17·1, 22·1	22·5	19·2, 25·8	2·9	–1·3, 7·0	11·1	9·1, 13·1	12·1	9·5, 14·7	1·0	–2·2, 4·3
25–34	16·3	14·5, 18·1	22·8	20·5, 25·1	6·5	3·6, 9·4	8·8	7·5, 10·2	12·5	10·8, 14·2	3·6	1·5, 5·8
35–44	14·8	13·0, 16·6	20·2	17·9, 22·4	5·4	2·5, 8·2	8·6	7·1, 10·1	11·5	9·8, 13·3	2·9	0·6, 5·3
45–54	14·2	12·2, 16·1	19·0	16·6, 21·5	4·9	1·7, 8·0	6·8	5·5, 8·2	8·7	6·9, 10·5	1·9	–0·4, 4·1
55–64	9·6	8·0, 11·3	22·3	20·1, 24·5	12·6	9·9, 15·4	4·7	3·4, 5·9	9·6	8·0, 11·2	4·9	2·9, 7·0
65–74	10·5	8·7, 12·4	20·1	17·6, 22·7	9·6	6·5, 12·8	3·6	2·5, 4·7	8·4	6·6, 10·1	4·8	2·7, 6·8
75+	9·2	5·2, 13·2	14·4	10·0, 18·8	5·2	–0·7, 11·1	2·1	0·4, 3·8	6·5	3·6, 9·4	4·5	1·1, 7·8
Education												
Low	14·7	13·1, 16·2	18·7	17·1, 20·4	4·1	1·8, 6·3	7·0	5·8, 8·1	8·8	7·6, 10·0	1·8	0·2, 3·5
Medium	13·6	12·2, 15·0	21·6	19·8, 23·4	8·0	5·7, 10·3	6·0	4·9, 7·0	10·0	8·7, 11·4	4·0	2·4, 5·7
High	13·5	12·4, 14·5	24·2	22·6, 25·8	10·7	8·9, 12·6	8·0	7·2, 8·8	12·9	11·7, 14·1	4·9	3·5, 6·4

**Table 6:** Predicted probability weighted estimates – % (95 % CI) – for models tested with interaction between policy and sociodemographic variables

	Ordered something different					
	No policy%	95 % CI	Policy present%	95 % CI	Difference%	95 % CI
Ethnicity						
Majority	16·3	15·5, 17·2	21·0	19·9, 22·1	4·7	3·3, 6·1
Minority	24·0	21·3, 26·6	19·1	17·2, 21·0	−4·8	–8·0, –1·6

**Table 7:** Predicted probability weighted estimates – % (95 % CI) – for models tested with interaction between policy and sociodemographic variables

	Changed restaurant visited					
	No Policy%	95 % CI	Policy Present%	95 % CI	Difference%	95 % CI
Education						
Low	7·1	5·9, 8·2	6·1	5·2, 7·1	−0·9	–2·4, 0·6
Medium	6·4	5·4, 7·4	9·5	8·2, 10·9	3·1	1·4, 4·8
High	7·4	6·6, 8·2	9·7	8·7, 10·7	2·3	1·0, 3·6
Income Adequacy						
Not Easy	7·0	6·3, 7·7	7·3	6·6, 8·1	0·3	–0·8, 1·4
Easy	6·8	5·8, 7·8	9·7	8·6, 10·9	2·9	1·4, 4·4

For *ordered something different*, the differences between policy groups were directionally different for the majority ethnicity group (difference of 4·7 %, 95 % CI 3·3, 6·1; *P* < 0·001) compared to the minority ethnicity group (difference of –4·8 %, 95 % CI –8·0 to, 1·6; *P* < 0·001). These differences were primarily due to high rates of *ordering something different* for minority groups in No Policy jurisdictions (Table 6).

For *changed restaurant visited*, the difference between policy groups was greater for medium (difference of 3·1 %, 95 % CI 1·4, 4·8, *P* < 0·001) and high education (difference of 2·3 %, 95 % CI 1·0, 3·6, *P* < 0·01) compared to low education (difference of –0·9 %, 95 % CI –2·4, 0·6) (Table 7). The difference between policy groups was greater for higher income adequacy (difference of 2·9 %, 95 % CI 1·4, 4·4) compared to lower income adequacy (difference of 0·3 %, 95 % CI –0·8, 1·4) (Table 7).

## Discussion

To our knowledge, this is the first multi-country examination of national and state/province-level menu labelling policies and associated behaviours. In this online multi-country survey with 19 393 participants conducted in 2019, we find evidence that implementation of mandatory menu labelling in restaurants is associated with a range of behaviours that are on the pathway from exposure to mandatory menu labelling to change in individual purchasing and eating. When differences by sociodemographic factors were present, the greatest differences were seen in those of middle to older age and those with greater socio-economic affluence according to education or perceived income adequacy.

### Interpretation and implications of findings

Our study builds on previous work measuring noticing and using nutrition information with a multi-country comparison of jurisdictions where energy labelling on menus was mandatory compared to where it was not. Jurisdictions with mandatory nutrition labelling policies had higher rates of noticing nutrition information, ordering something different, eating less of what was ordered and changing restaurants due to nutrition information availability. However, there were no differences between policy groups in frequency of eating out at restaurants. This suggests menu labels may affect behaviour within restaurants, but do not affect consumers’ decision of whether or not to eat at a restaurant.

Most importantly, these results suggest that mandatory menu labelling policies may be improving behaviours associated with menu labelling at restaurants when comparing jurisdictions with and without mandatory menu labelling policies. These changes are according to our proposed mechanism involving noticing, using and types of use all leading to changes in energy consumed. There is evidence for the link between noticing labels and behaviour change. For example, noticing other types of labels such as traffic light labels has been found to be associated with healthier items purchased^(39)^. Mandatory menu labelling policies have increased noticing and use of nutrition information in other contexts, but more evidence is needed to understand whether these findings are consistent for older age groups and in other countries and population subgroups^(33)^. More recent evidence from the USA suggests the small-to-moderate reductions in energy purchased may diminish over time, potentially reducing the long term public health impact of energy labels. The present study found significant differences between mandatory and non-mandatory menu labelling jurisdictions for five out of six behavioural outcomes measured, which could potentially lead to improved diets across large populations.

Although noticing and use of nutrition information was greater in jurisdictions with mandatory labelling policies, estimates were relatively low (21·2 % noticing and 10·6 % using nutrition information), and the differences in behaviours associated with menu labelling are modest. However, these differences could still be meaningful for health when they include millions of people. To improve public health, there may be ways to augment the effects of menu labelling policies. First, menu labelling interventions may be optimised by further helping people notice nutrition information – for example by making the information more prominent via increased size or visual salience^(40)^. Second, interventions could help people use nutrition information by including associated messaging such as choosing an option with lower energy content to benefit health. For example, evaluative labels may be easier to interpret than numerical labels^(41)^, and adding a recommended daily energy intake alongside menu labels maybe increase their effects^(42)^. Previous work has also found that motivation to use nutrition information may be a more important barrier than mere nutrition knowledge^(43)^. Third, additional policies are needed to have a large impact on diets across populations. Mandatory menu labelling may be a component of an effective obesity reduction strategy, but it is unlikely to achieve government targets to reduce obesity without complementary policies as eating behaviours are influenced by numerous complex factors beyond individual decision-making processes.

Although our study suggests mandatory menu labelling policies may play a role in reducing energy consumption out of home, other mechanisms of action may have more important effects, and mandatory labelling policies alone may not be enough to greatly reduce energy intake out of home across large populations. Additional messaging about how to use energy information alongside mandatory menu labelling policies may augment consumers noticing and ability to use this information to make healthier food choices when eating outside the home. People may also lack guidance regarding how to understand and use nutrition information to eat healthier and ultimately improve their health. Some jurisdictions such as New York City have tried to supplement mandated energy information posted on chain restaurant menus by adding recommended energy intake per day or per meal, but with no effect^(25)^. Future work is needed to determine whether other policies that reduce energy consumed out of home are more effective than menu labelling, and more research is needed to understand whether the sociodemographic differences in self-reported behaviours found in this study also exist for dietary intake. Menu labelling may also spur reformulation of products to lower energy or healthier forms by reducing nutrients of concern. However, energy labelling in large chain restaurants was associated with minimal changes in energy content of menu items, primarily consisting of the introduction of new lower energy items^(44)^.

### Differences between policy groups by sociodemographic characteristics

In addition to estimating the differences between policy groups for each of the six behavioural outcomes, we found several differences between policy groups by demographic characteristics. Examining differences between policy groups by age, the youngest age group, aged 18–24 were the mostly likely to eat less of their order compared to other age groups. Among United Kingdom diners at catering establishments, younger groups were more interested in menu labelling than older groups^(45)^, and this greater interest could translate into greater use. On the other hand, the greatest differences for noticing and using menu labels were found for the middle and upper-middle age groups. Noticing and using nutrition information were more common for younger age groups living in No Policy jurisdictions, and differences between policy groups were larger for older groups. This finding is supported by a systematic review of nutrition labels on pre-packaged foods that found older adults were less likely to use nutrition labels than middle-aged and young adults^(46)^. Our study similarly found low noticing and use of nutrition information among older age groups, but living in a Policy Present area reduced some of the disparity.

Examining the differences between policy groups by education, rates of noticing nutrition information, using nutrition information and changing restaurants were roughly equal in No Policy jurisdictions, but the increases were greater for higher education and majority ethnicity groups in Policy Present jurisdictions (Tables 5 and 6). Differences between policy groups for changing restaurants were also greater in the higher income adequacy group compared to the lower income adequacy group, and the higher income adequacy group was more likely to change restaurants in the Policy Present group (Table 7). This suggests that higher education and higher income groups may be more sensitive to changing restaurants within Policy Present jurisdictions. Indeed, higher comprehension and use of nutritional labels has been found to be associated with higher income and higher education^(46)^. Higher education and higher income levels were associated with a greater likelihood of ordering something different, changing restaurants and eating at restaurants less often. The highest income level group was also the most likely to eat less of what they ordered due to noticing nutrition information. This is concordant with the majority of previous evidence which also suggests higher education and income levels are associated with greater use of nutrition labels^(46)^. Although some other research has not found any convincing evidence of sociodemographic disparities in responses to menu labelling, the present results suggest that menu labelling policies, specifically those containing only numeric information, could potentially widen inequalities in healthy eating and therefore health^(47,48)^. This insight suggests that other interventions may be needed to support lower education groups through mechanisms other than information-based policies that require significant cognitive demand on individuals. There is evidence that labels can be designed to avoid widening disparities between socio-economic groups. Other types of labels, such as warning labels, may be of greater use for people of low income or literacy, thereby reducing disparities^(49)^. Therefore, our results suggest the association between label design and socio-economic disparities should be considered when designing labels for real world policies.

The difference between policy groups was greater in the majority ethnicity group compared to the minority ethnicity group for ordering something different, but these differences were primarily due to high rates of ordering something different for minority groups in No Policy jurisdictions (Table 5). The rates of ordering something different in No Policy jurisdictions for minority groups were closer to the rates found in Policy Present jurisdictions for both minority and majority groups. Previous work from the USA found Black or Hispanic participants were more likely to choose restaurants with menu labelling and to use caloric information compared to White participants^(50)^. A similar pattern could explain our findings of greater ordering something different in No Policy jurisdictions if minority groups are more likely to seek restaurants that have menu labelling compared to majority groups. These findings suggest that there may be limited additional benefit of mandatory menu labelling policies for minorities if they already using nutrition information at a greater rate than in voluntary jurisdictions. However, further work is needed to determine whether this pattern exists across other populations. Overall, these differential effects of menu labelling in restaurants across groups suggest complementary policies may be needed to support healthy eating and reduce inequalities across more vulnerable socio-economic groups.

### Strengths and limitations

To our knowledge, this is the first multi-country examination of national and state/province-level menu labelling policies and associated behaviours. Using the same survey questions across intervention and comparison policy jurisdictions allows for between-country comparisons that are otherwise more challenging to do between countries with limited capacity to conduct routine national diet surveys^(27)^. Thus, these results may help provide more generalisable evidence for the effects of menu labelling on self-reported eating behaviours. Weighted IFPS estimates are close to the sociodemographic distributions in the countries studied, although there was a lower recruitment of low education participants from Mexico^(27)^. Finally, the large study sample of nearly twenty thousand participants increases power to detect differences between policy groups.

Our study does have limitations as we cannot determine the degree to which self-reported behavioural changes translate to changes in dietary intake, obesity or other health outcomes. There are also some variations in menu labelling policies within our Policy Present and No Policy groups. Although we were able to categorise jurisdictions into either Policy Present or No Policy groups, the mandatory policies in Canada, Australia and the USA are not exactly the same – for example, businesses with twenty or more locations in Ontario, Canada, could refer to businesses with greater density of outlets compared to with twenty or more locations across the USA, given the different density of outlets required to meet the twenty outlet threshold in geographic areas of different size. Thus, our study has some challenges to consistency – variations of exposure (Policy Present in this study) do not differentially affect outcomes (behavioural measures in this study) – a core assumption of causal inference. The cross-sectional nature of a single data collection and a natural experimental design are more vulnerable to confounding bias than randomised controlled trials for demonstrating causal effects, and we cannot eliminate the possibility for residual confounding if some factor other than policy status is driving the differences between groups, such as country differences. Due to the single year of data used, we also cannot determine whether differences observed in 2019 are due to reverse causation: for example, if pre-policy rates of noticing and using menu labels were higher and thereby facilitated policy adoption. Finally, we did not examine all mechanisms through which menu labels could improve population health. Menu labels could improve health through pathways other than the behaviours examined here – for example through product reformulation – which could reduce energy intakes.

### Conclusions

Participants living in jurisdictions with mandatory nutrition information were more likely to report noticing nutrition labels, ordering something different, eating less of what was ordered, and changing restaurants in jurisdictions where nutrition information in restaurants was mandatory. The magnitudes of differences between Policy and No Policy jurisdictions were relatively small. Mandatory menu labelling was associated with greater behavioural differences in more socio-economically affluent groups, which could potentially exacerbate existing inequalities in diet and health. Complementary interventions may be required to optimise mandatory menu labelling interventions by accounting for unequal effects across sociodemographic groups. Further research understanding whether menu labelling has similar inequitable effects on dietary intake will now be valuable.

## Supplementary material

For supplementary material accompanying this paper visit https://doi.org/10.1017/S1368980023001775. click here to view supplementary material

## References

[1] World Health Organisation Regional Office for Europe (2021) The Out-of-Home Food Sector – Exponential Growth in an Unregulated Market. https://www.who.int/europe/news/item/20-09-2021-the-out-of-home-food-sector-exponential-growth-in-an-unregulated-market (accessed June 2022).

[2] Lachat C , Nago E , Verstraeten R et al. (2012) Eating out of home and its association with dietary intake: a systematic review of the evidence. Obes Rev 13, 329–346. 22106948 10.1111/j.1467-789X.2011.00953.x

[3] Nago ES , Lachat CK , Dossa RAM et al. (2014) Association of out-of-home eating with anthropometric changes: a systematic review of prospective studies. Crit Rev Food Sci Nutr 54, 1103–1116. 24499144 10.1080/10408398.2011.627095

[4] Gesteiro E , García-Carro A , Aparicio-Ugarriza R et al. (2022) Eating out of home: influence on nutrition, health, and policies: a scoping review. Nutrients 14, 1–15. 10.3390/nu14061265 PMC8953831 35334920

[5] Livingstone MBE & Pourshahidi LK (2014) Portion size and obesity. Adv Nutr 5, 829–834. 25398749 10.3945/an.114.007104 PMC4224223

[6] Block JP , Condon SK , Kleinman K et al. (2013) Consumers’ estimation of calorie content at fast food restaurants: cross sectional observational study. BMJ 346, 1–10. 10.1136/bmj.f2907 PMC3662831 23704170

[7] VanEpps EM , Roberto CA , Park S et al. (2016) Restaurant menu labeling policy: review of evidence and controversies. Curr Obes Rep 5, 72–80. 26877095 10.1007/s13679-016-0193-z PMC5124489

[8] Kaur A , Briggs A , Adams J et al. (2022) New calorie labelling regulations in England. BMJ 377, o1079. 35508318 10.1136/bmj.o1079

[9] WHO (2017) ‘Best buys’ and other recommended interventions for the prevention and control of noncommunicable diseases. World Health Organ 17, 28.

[10] Crockett RA , King SE , Marteau TM et al. (2018) Nutritional labelling for healthier food or non-alcoholic drink purchasing and consumption. Cochrane Database Syst Rev issue 2, CD009315. 10.1002/14651858.CD009315.pub2 PMC5846184 29482264

[11] Agarwal D , Ravi P , Purohit B et al. (2022) The effect of energy and fat content labeling on food consumption pattern: a systematic review and meta-analysis. Nutr Rev 80, 453–466. 34339509 10.1093/nutrit/nuab035

[12] Littlewood JA , Lourenço S , Iversen CL et al. (2016) Menu labelling is effective in reducing energy ordered and consumed: a systematic review and meta-analysis of recent studies. Public Health Nutr 19, 2106–2121. 26714776 10.1017/S1368980015003468 PMC10270829

[13] Bleich SN , Economos CD , Spiker ML et al. (2017) A systematic review of calorie labeling and modified calorie labeling interventions: impact on consumer and restaurant behavior. Obesity 25, 2018–2044. 29045080 10.1002/oby.21940 PMC5752125

[14] Sinclair SE , Cooper M & Mansfield ED (2014) The influence of menu labeling on calories selected or consumed: a systematic review and meta-analysis. J Acad Nutr Diet 114, 1375–1388. 25037558 10.1016/j.jand.2014.05.014

[15] Long MW , Tobias DK , Cradock AL et al. (2015) Systematic review and meta-analysis of the impact of restaurant menu calorie labeling. Am J Public Health 105, e11–e24. 10.2105/AJPH.2015.302570 PMC4386504 25790388

[16] Harnack LJ & French SA (2008) Effect of point-of-purchase calorie labeling on restaurant and cafeteria food choices: a review of the literature. Int J Behav Nutr Phys Act 5, 3–8. 18950529 10.1186/1479-5868-5-51 PMC2584065

[17] Kiszko K , Martinez O , Abrams C et al. (2014) The influence of calorie labeling on food orders and consumption: a review of the literature. J Community Health 39, 1248–1269. 24760208 10.1007/s10900-014-9876-0 PMC4209007

[18] Cantu-Jungles TM , McCormack LA , Slaven JE et al. (2017) A meta-analysis to determine the impact of restaurant menu labeling on calories and nutrients (Ordered or consumed) in U.S. adults. Nutrients 9, 20–22. 28973989 10.3390/nu9101088 PMC5691705

[19] Petimar J , Zhang F , Rimm EB et al. (2021) Changes in the calorie and nutrient content of purchased fast food meals after calorie menu labeling: a natural experiment. PLoS Med 18, 1–17. 10.1371/journal.pmed.1003714 PMC8312920 34252088

[20] Petimar J , Zhang F , Cleveland LP et al. (2019) Estimating the effect of calorie menu labeling on calories purchased in a large restaurant franchise in the southern United States: quasi-experimental study. BMJ 367, I5837. 10.1136/bmj.l5837 PMC6818731 31666218

[21] Petimar J , Grummon AH , Zhang F et al. (2022) Assessment of calories purchased after calorie labeling of prepared foods in a large supermarket chain. JAMA Intern Med 182, 965–973. 35913728 10.1001/jamainternmed.2022.3065 PMC9344388

[22] Fernandes AC , Oliveira RC , Proenca RPC et al. (2016) Influence of menu labeling on food choices in real-life settings: a systematic review. Nutr Rev 74, 534–548. 27358442 10.1093/nutrit/nuw013

[23] Elbel B , Mijanovich T , Dixon B et al. (2013) Calorie labeling, fast food purchasing and restaurant visits. Obesity (Silver Spring) 21, 2172–2179. 24136905 10.1002/oby.20550 PMC3947482

[24] Vadiveloo MK , Dixon LB & Elbel B (2011) Consumer purchasing patterns in response to calorie labeling legislation in New York City. Int J Behav Nutr Phys Act 8, 51. 21619632 10.1186/1479-5868-8-51 PMC3123618

[25] Downs JS , Wisdom J , Wansink B et al. (2013) Supplementing menu labeling with calorie recommendations to test for facilitation effects. Am J Public Health 103, 1604–1609. 23865657 10.2105/AJPH.2013.301218 PMC3780676

[26] Micha R , Coates J , Leclercq C et al. (2018) Global dietary surveillance: data gaps and challenges. Food Nutr Bull 39, 175–205. 29478333 10.1177/0379572117752986

[27] Hammond D , Vanderlee L , White CM et al. (2022) The conceptual framework for the international food policy study: evaluating the population-level impact of food policy. J Nutr 152, 1S–12S. 35274695 10.1093/jn/nxac042 PMC9188864

[28] Sofía Rincón-Gallardo P , Zhou M , Gomes FDS et al. (2020) Effects of menu labeling policies on transnational restaurant chains to promote a healthy diet: a scoping review to inform policy and research. Nutrients 12, 1–27. 10.3390/nu12061544 PMC7352298 32466387

[29] Hammond D , White C , Rynard V et al. (2021) Technical Report 2019 Survey (Wave 3). International Food Policy Study. www.foodpolicystudy.com/methods (accessed June 2022).

[30] legislation.gov.uk (2021) The Calorie Labelling (Out of Home Sector) (England) Regulations. https://www.legislation.gov.uk/uksi/2021/909/made (accessed June 2022).

[31] Willmott TJ , Pang B & Rundle-Thiele S (2021) Capability, opportunity, and motivation: an across contexts empirical examination of the COM-B model. BMC Public Health 21, 1–17. 34051788 10.1186/s12889-021-11019-w PMC8164288

[32] Roberto CA & Kawachi I (2014) Use of psychology and behavioral economics to promote healthy eating. Am J Prev Med 47, 832–837. 25441239 10.1016/j.amepre.2014.08.002

[33] Goodman S , Vanderlee L , White CM et al. (2018) A quasi-experimental study of a mandatory calorie-labelling policy in restaurants: impact on use of nutrition information among youth and young adults in Canada. Prev Med 116, 166–172. 30261242 10.1016/j.ypmed.2018.09.013

[34] Litwin H & Sapir EV (2009) Perceived income adequacy among older adults in 12 countries: findings from the survey of health, ageing, and retirement in Europe. Gerontologist 49, 397–406. 19386829 10.1093/geront/gnp036 PMC2682171

[35] Williams R (2012) Using the margins command to estimate and interpret adjusted predictions and marginal effects. Stata J 12, 308–331.

[36] Mood C (2010) Logistic regression: why we cannot do what we think we can do, and what we can do about it. Eur Sociol Rev 26, 67–82.

[37] Norton EC , Dowd BE & Maciejewski ML (2019) Marginal effects—quantifying the effect of changes in risk factors in logistic regression models. JAMA 321, 1304–1305. 30848814 10.1001/jama.2019.1954

[38] StataCorp (2019) *Stata Statistical* Software*: Release 16*. College Station, TX: StataCorp; available at https://www.stata.com/manuals/rpwcompare.pdf (accessed August 2022).

[39] Sonnenberg L , Gelsomin E , Levy D et al. (2013) A traffic light food labeling intervention increases consumer awareness of health and healthy choices at the point-of-purchase. Prev Med 57, 253–257. 23859926 10.1016/j.ypmed.2013.07.001 PMC3913274

[40] Peschel AO , Orquin JL & Mueller Loose S (2019) Increasing consumers’ attention capture and food choice through bottom-up effects. Appetite 132, 1–7. 30248439 10.1016/j.appet.2018.09.015

[41] Thorndike AN , Sonnenberg L , Riis J et al. (2012) A 2-phase labeling and choice architecture intervention to improve healthy food and beverage choices. Am J Public Health 102, 527–533. 22390518 10.2105/AJPH.2011.300391 PMC3329221

[42] Roberto CA , Larsen PD , Agnew H et al. (2010) Evaluating the impact of menu labeling on food choices and intake. Am J Public Health 100, 312–318. 20019307 10.2105/AJPH.2009.160226 PMC2804627

[43] Grunert KG , Fernández-Celemín L , Wills JM et al. (2010) Use and understanding of nutrition information on food labels in six European countries. J Public Health 18, 261–277. 21124644 10.1007/s10389-009-0307-0 PMC2967247

[44] Grummon AH , Petimar J , Soto MJ et al. (2021) Changes in calorie content of menu items at large chain restaurants after implementation of calorie labels. JAMA Netw Open 4, 1–12. 10.1001/jamanetworkopen.2021.41353 PMC8719240 34967879

[45] Mackison D , Wrieden W & Anderson A (2009) Making an informed choice in the catering environment: what do consumers want to know? J Hum Nutr Diet 22, 567–573. 20002952 10.1111/j.1365-277X.2009.01000.x

[46] Campos S , Doxey J & Hammond D (2011) Nutrition labels on pre-packaged foods: a systematic review. Public Health Nutr 14, 1496–1506. 21241532 10.1017/S1368980010003290

[47] Robinson E , Polden M , Langfield T et al. (2023) Socioeconomic position and the effect of energy labelling on consumer behaviour: a systematic review and meta-analysis. Int J Behav Nutr Phys Act 20, 1–15. 36747247 10.1186/s12966-023-01418-0 PMC9903416

[48] Robinson E , Boyland E , Christiansen P et al. (2023) Is the effect of menu energy labelling on consumer behaviour equitable? A pooled analysis of twelve randomized control experiments. Appetite 182, 106451. 36610541 10.1016/j.appet.2023.106451 PMC10082393

[49] Hall MG , Lazard AJ , Grummon AH et al. (2021) Designing warnings for sugary drinks: a randomized experiment with Latino parents and non-Latino parents. Prev Med 148, 1–18. 10.1016/j.ypmed.2021.106562 PMC8958622 33878350

[50] Bleich SN & Pollack KM (2010) The publics’ understanding of daily caloric recommendations and their perceptions of calorie posting in chain restaurants. BMC Public Health 10, 121. 20214811 10.1186/1471-2458-10-121 PMC2847976

