# Supplementary material for: A multi-country comparison of jurisdictions with and without mandatory nutrition labelling policies in restaurants: analysis of behaviours associated with menu labelling in the 2019 International Food Policy Study
Source: Public Health Nutr. 2023 Sep 4;26(11):2595–606. doi: 10.1017/S1368980023001775 (PMC10641604; doi:10.1017/S1368980023001775)
Supplement: Supplementary file 1 [file S1368980023001775sup001.docx]

**Supplementary Materials**

**Supplementary Table 1**. Unweighted prevalence of noticing, using, and behavior change analyzed by country and policy status

| **Country** | **Noticed Nutrition Info**  **n (%)** | | **Used Nutrition Info**  **n (%)** | | **Ordered Something Different**  **n (%)** | | **Ate Less**  **n (%)** | | **Changed Restaurant**  **n (%)** | | **Restaurants Less Often**  **n (%)** | |
| --- | --- | --- | --- | --- | --- | --- | --- | --- | --- | --- | --- | --- |
|  | No | Yes | No | Yes | No | Yes | No | Yes | No | Yes | No | Yes |
| **Australia – Policy*** | 2970 | 417 | 3191 | 196 | 2893 | 494 | 3039 | 348 | 3125 | 262 | 2938 | 449 |
| n=3387 | (87.7) | (12.3) | (94.2) | (5.8) | (85.4) | (14.6) | (89.7) | (10.3) | (92.3) | (7.7) | (86.7) | (13.3) |
| **Australia - No Policy** | 412 | 54 | 444 | 22 | 390 | 76 | 419 | 47 | 435 | 31 | 404 | 62 |
| n=466 | (88.4) | (11.6) | (95.3) | (4.7) | (83.7) | (16.3) | (89.0) | (10.1) | (93.3) | (6.7) | (86.7) | (13.3) |
| **Canada – Policy*** | 911 | 417 | 1141 | 187 | 1003 | 325 | 1151 | 177 | 1211 | 117 | 1112 | 216 |
| n=1328 | (68.6) | (31.4) | (85.9) | (14.1) | (75.5) | (24.5) | (86.7) | (13.3) | (91.2) | (8.8) | (83.7) | (16.3) |
| **Canada - No Policy** | 2144 | 375 | 2358 | 161 | 2132 | 387 | 2254 | 265 | 2358 | 161 | 2095 | 424 |
| n=2519 | (85.1) | (14.9) | (93.6) | (6.4) | (84.6) | (15.4) | (89.5) | (10.5) | (93.6) | (6.4) | (83.2) | (16.8) |
| **Mexico – No Policy** | 3588 | 459 | 3737 | 310 | 3033 | 1014 | 3617 | 430 | 3736 | 311 | 3306 | 741 |
| n=4047 | (88.7) | (11.3) | (92.3) | (7.7) | (74.9) | (25.1) | (89.4) | (10.6) | (92.3) | (7.7) | (81.7) | (18.3) |
| **UK – No Policy** | 3097 | 608 | 3435 | 270 | 3186 | 519 | 3342 | 363 | 3460 | 245 | 3338 | 367 |
| n=3705 | (83.6) | (16.4) | (92.7) | (7.3) | (86.0) | (14.0) | (90.2) | (9.8) | (93.4) | (6.6) | (90.1) | (9.9) |
| **USA – Policy** | 2887 | 1054 | 3363 | 578 | 2956 | 985 | 3324 | 617 | 3595 | 346 | 3355 | 586 |
| n=3941 | (73.3) | (26.7) | (85.3) | (14.7) | (75.0) | (25.0) | (84.3) | (15.7) | (91.2) | (8.8) | (85.1) | (14.9) |

*Australia and Canada are separated into policy/no policy groups according to which jurisdictions have mandatory calorie labelling in restaurants. These policy differences are described in Table 1.
